# Assessment of Atg7 and LC3II/LC3, as The Markers of Autophagy, in Sperm of Infertile Men with Globozoospermia: A Case-Control Study 

**DOI:** 10.22074/cellj.2021.7023

**Published:** 2021-03-01

**Authors:** Shaghayegh Foroozan-Boroojeni, Marziyeh Tavalaee, Zahra Zakeri, Richard A. Lockshin, Mohammad Hossein Nasr-Esfahani

**Affiliations:** 1. Department of Animal Biotechnology, Reproductive Biomedicine Research Center, Royan Institute for Biotechnology, ACECR, Isfahan, Iran; 2. Department of Biology, Queens College and Graduate Center of The City University of New York, Flushing, NY, USA; 3. Department of Biological Sciences, St. John’s University, Jamaica, NY, USA; 4. Isfahan Fertility and Infertility Center, Isfahan, Iran

**Keywords:** Acrosome, Autophagy, Chromatin, Globozoospermia, Infertility

## Abstract

**Objective:**

Assessment of relationship between LC3II/LC3 and Autophagy-related 7 (Atg7) proteins, as markers of autophagy, as well as evaluating the sperm parameters and DNA fragmentation in spermatozoa of infertile men with globozoospermia.

**Materials and Methods:**

In this case-control study, 10 semen samples from infertile men with globozoospermia and 10 fertile individuals were collected, and the sperm parameters, sperm DNA fragmentation, and main autophagy markers (Atg7 and LC3II/LC3) were assessed according to World Health Organization (WHO) criteria, TUNEL assay, and western blot technique, respectively.

**Results:**

The mean of sperm concentration and motility were significantly lower, while the percentage of abnormal spermatozoa and DNA fragmentation were significantly higher in infertile men with globozoospermia compared to fertile individuals (P<0.01). Unlike the relative expression of LC3II/LC3 that did not significantly differ between the two groups, the relative expression of ATG7 was significantly higher in infertile men with globozoospermia compared to fertile individuals (P<0.05). There was a significantly negative correlation between the sperm concentration (r=-0.679; P=0.005) and motility (r=-0.64; P=0.01) with the expression of ATG7, while a significantly positive association was founf between the percentage of DNA fragmentation and expression of ATG7 (0.841; P =0.018).

**Conclusion:**

The increased expression of ATG7 and unaltered expression of LC3II/LC3 may indicate that the autophagy pathway is initiated but not completely executed in spermatozoa of individuals with globozoospermia. A significant correlation of ATG7 expression with increased sperm DNA fragmentation, reduced sperm concentration, and sperm motility may associate with the activation of a compensatory mechanism for promoting deficient spermatozoa to undergo cell death by the autophagy pathway. Therfore, this pathway could act as a double-edged sword that, at the physiological level, is involved in acrosome biogenesis, while, at the pathological level, such as globozoospermia, could act as a compensatory mechanism.

## Introduction

Round-headed sperm syndrome or globozoospermia, is one of the types of monomorphic severe teratozoospermia (abnormal spermatozoa that are defective in terms of function and morphology), leading to a decrease or an absence of the acrosome in sperm cells. It is also characterized by the abnormal arrangement of mitochondria, aberrant nuclear membrane, and mid-piece defects (1,2). The absence of acrosome and lack of ability of these sperm to induce oocyte activation are considered the main reason for infertilization of these patients (3).

Globozoospermia has a genetic mode of inheritance as autosomal recessive (4). In this regard, three genes were identified related to globozoospermia namely,* SPATA16, PICK1*, and *DPY19L2* (5-7). These genes are somehow associated with the formation and localization of the acrosome (7). In animal models, it is now known that the absence of the expression of other genes, such as Autophagy-related 7 (*Atg7*), HIV-1 Rev-binding protein (*HRB*), Golgi-associated PDZ, coiled-coil motif containing protein (*GOPC*), casein kinase 2, and α-prime polypeptide (*CSNK2A2*) induce a phenotype similar to globozoospermia (7-9). All of these genes are linked to biogenesis of the acrosome; the development of which commences with the formation and fusion of many pro-acrosomic granules from trans-Golgi stacks to create a single large acrosomic granule binding to the nucleus and subsequently covering this structure to form a mature acrosome (7). In addition, autophagy is also essential for acrosomal biogenesis, and the inactivation of testicular Atg7 leads to deformity in acrosome structure (8).

Autophagy is considered one of the main intracellular process that can degrade and/or recycle long-lived proteins and organelles. From a molecular point of view, Atg12-Atg5 and LC3-lipid/membrane, as two ubiquitin-like conjugation systems, form the core of autophagy machinery (10). For both of these conjugation systems, Atg7 is a key molecule, as it is able to activate both systems. The outcome of these molecular events can be observed at cellular levels, as the engulfment of cytoplasmic components is mediated by a doublemembrane vesicle known as autophagosomes. The fusion of autophagosomes with lysosomes ultimately creates autolysosome. The autophagosome membrane, along with its contents is subseqyently degraded by hydrolases enzymes, residing in autolysosome (10).

Considering autophagy plays a dual role in cell death and acrosomal biogenesis, and also, spermatozoa of infertile men with globozoospermia have low or no acrosome, we aimed to assess the two central markers of autophagy, namely Atg7 and LC3II/LC3, as well as the sperm parameters and DNA fragmentation in these individuals.

## Materials and Methods

This case-control study was approved by the review board of the Royan Institute (Ethical Code: IR.ACECR. ROYAN.REC.1397.15). The written informed consent was obtained from all individuals who participated in this study .Semen samples were collected from subjects who referred to Isfahan Fertility and Infertility Center. Totally 10 ,semen samples from infertile men with globozoospermia and 10 semen samples from fertile men were collected for this study .All globozoospermic samples had DPY19L2 deletion that was assessed according to our previous study (6). Couplese with female factor infertility and men with leukocytospermia, genital infection, anatomical disorders, abnormal hormonal profle, varicocele, previous history of scrotal trauma or surgery, and age >40 years were excluded from this study. 

### Semen samples collection

Semen samples were collected after 3-5 days of sexual abstinence by masturbation in the sterile containers and kept for 30 minutes at room temperature to liquefy. In the first step, the sperm parameters (concentration, motility, and morphology) were evaluated according to guidelines provided by WHO (11). The sperm concentration and motility were determined by CASA (computer-assessed semen analysis; version Sperm 2.1# 1990-2004, Russia) system secured with a sperm processor chamber (Sperm meter; Sperm Processor, Aurangabad, India). Besides, sperm morphology was assessed by the Diff-Quick staining method and analyzed by CASA system. In the second step, we evaluated DNA fragmentation by the TUNEL assay and authophagy markers (Atg7 and LC3II/LC3I) by the western blot technique.

### Diff-Quick staining

**Fig.1:**
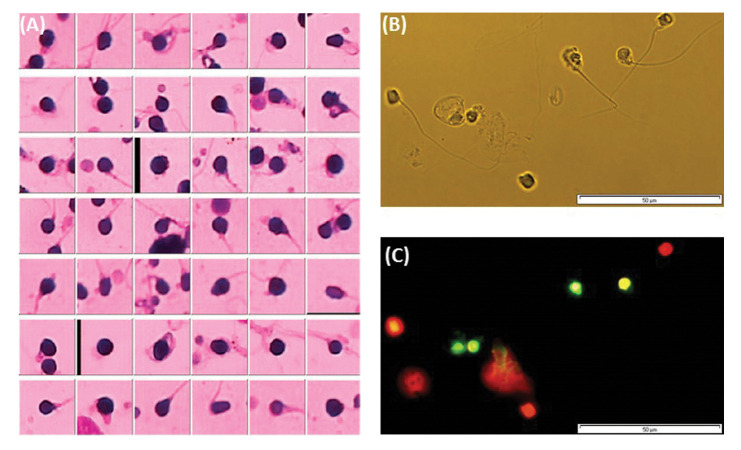
Assessment of sperm morphology and DNA fragmentation using Diff-Quick staining and TUNEL assay, respectively. **A. **Sperm sample from an infertile men with globozoospermia was satined with DiffQuick method for assessment of sperm morphology and analyzed by CASA system (computer-assessed semen analysis; version Sperm 2.1# 1990-2004, Russia). **B** and **C. **Sperm sample from an infertile men with globozoospermia was assessed by TUNEL assay for evaluation of sperm DNA fragmentation (B: Light microscop; C: Floresecnce microscope).

For the assessment of sperm morphology, we purchased a commercially available kit (Faradidpardaz Co, Iran), containing a fixative soloution (methanol), eosin dye for staining basic proteins (red), and thiazine dye for staining sperm DNA (blue). Briefly, 20 microlitres of the washed samples were smeared and air-dried. Then, slides were sequentially soaked in fixative, eosin, and thiazine solutions for 10-20 seconds, and finally rinsed in water to remove extra dye (11). Next, abnormalities in head, tail, acrosome, and neck of spermatozoa were evaluated (Fig .1A).

### TUNEL assay

In this study, we determined DNA fragmentation of spermatozoa using a detection kit (Apoptosis Detection System Fluorescein, Promega, Germany), according to the manufacturer’s instructions. Briefly, semen samples were washed twice with phosphate-buffered solution (PBS), and then for each sample, two smears were prepared. Then, slides were fixed in 4% paraformaldehyde for 25 minutes. Subsequently, slides were washed with PBS. Fixed spermatozoa were permeabilizaed with 0.2% Triton X-100 for 5 minutes. The next step was equilibration of slides with equilibration buffer for 7 minutes, and then incubation of slides with a mixture soloution, containing nucleotide mix, rTdT, and equilibration buffer for 90 minutes at 37˚C in a humidified chamber. Lastly, reactions were stopped by 2X SSC buffer and then the slides were washed with PBS. We stained slides with a freshly diluted propidium iodide solution (1 μg/ml in PBS) for 10 minutes and then slides were washed in PBS. For the evaluation of the percentage of DNA fragmentation, we used a fluorescence microscope (BX51, Tokyo, Japan). At least, 500 spermatoza were counted for each sample. Spermatozoa with red nuclei were considered sperm with intact DNA, while those with green nuclei had damaged DNA, and they were reported as TUNEL-positive or DNA fragmented cells (Fig.1B and C). 

### Western blot

Protein extraction was performed from washed samples using TRI Reagent (Sigma-Aldrich; USA). Then, the protein concentration of each sample was evaluated by the Bradford assay (Bio-Rad; USA) to determine the amount of total proteins that should be loaded in each lane. For this aim, 40μg of protein of each sample were electrophoresed on 12% sodium dodecyl sulfate (SDS) polyacrylamide gels and then, the separated proteins were transferred onto PVDF membranes (Bio-Rad; USA). The membranes were blocked by PBS containing 5% skim milk powder (Merck, USA). For the detection of Atg7, LC3, and housekeeping glyceraldehyde-3-phosphate dehydrogenase (GAPDH), we used anti-Atg7 rabbit polyclonal antibody from Abcam company (Cambridge, MA, USA) with a dilution ratio of 1:1000, anti-LC3 rabbit polyclonal antibody from Novus Biologicals company (Littleton, CO, USA) with a dilution ratio of 1:4000, and monoclonal anti-glyceraldehyde GAPDH from Millipore company (USA) with a dilution ratio of 1:5000 as specific primary antibodies. For the first two proteins, membranes were incubated with proimary antibidies overnight, while for the dtermination of the GAPDH protein, the membrane was incubated with the specific primary antibody for 90 minutes. Afterward, membranes were washed and incubated for an hour with appropriate secondary antibodies. For tracking anti-Atg7 and antiLC3 antibodies, horseradish peroxidase (HRP) conjugated anti-rabbit IgG (Dako, Japan) was applied, while for probing the GAPDH protein, horseradish peroxidase (HRP)-conjugated goat anti-mouse IgG (Dako, Japan) was utilized as secondry antibodies. Then, membranes were rinsed three times. The presence of specific proteins was identified using an Amersham ECL Advance Western Blotting Detection Kit (GE Healthcare, Germany). For the quantification of data, the density of protein bands was analyzed by Quantity One 1-D Analysis software v 4.6.9 (Bio-Rad, Munchen, Germany). Normalization of data was performed by dividing of band densities of Atg7 and LC3 to the band density of Glyceraldehyde GAPDH, and represented as the expression level of Atg7 and LC3. Moreover, the ratio of LC3-II level/LC3-I level was measured as an indicator of autophagic level (12).

### Statistical analysis

For the comparison of the sperm parameters, DNA fragmentation, the expression of ATG7 and LC3II/LC3 proteins between fertile and globozoospermic men, independent t test was used. In this study, the values were expressed as the means and standard error (mean ± SE). The P values of less than 0.05 were statistically significant. For the evaluation of the relasionship between different parameters, pearson correlation coefficient was employed. All statistical analyses were conducted using the SPSS software (V19.0; IDM, Chicago, IL, USA).

## Results

The mean sperm concentrations (38.62 ± 8.63 vs. 82.64 ± 11.41; P=0.005) and sperm motility (32.85 ± 6.05 vs. 62 ± 4.27; P= 0.001) were significantly lower in infertile globozoospermic men compared to fertile men. The mean percentage of abnormal sperm morphology (100 ± 0.01 vs. 91.55 ± 2.44; P=0.006) was significantly higher in men with globozoospermia compared to fertile individuals. Also, the mean percentage of DNA fragmentation was significantly higher in men with globozoospermia compared to fertile individuals (18.18 ± 7.8 vs. 5.35 ± 1.9; P<0.05). 

**Fig.2:**
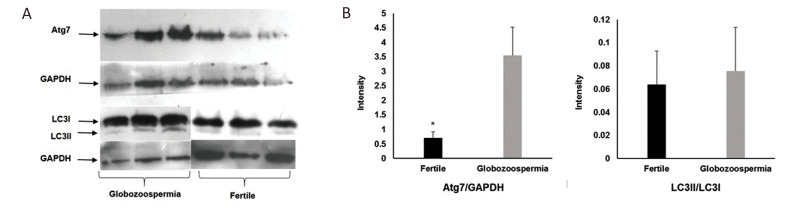
Western blot of autophagy markers Atg7 and LC3. **A.** Western blot image of three fertile individuals and three infertile men with globozoospermia. **B.** Intensity of Atg7 relative to GAPDH and LC3II/LC3I ratio between infertile men with globozoospermia and fertile individuals. Data are presented as mean ± SEM and analyzed by independent-samples t test (*P<0.05). GAPDH; Glyceraldehyde 3-phosphate dehydrogenase; and Atg7; Autophagy-related 7.

The role of autophagy flux in globozoospermia was studied by the western blot analysis to detect ATG7 and LC3II/LC3 proteins. The relative expression of ATG7 was significantly higher in infertile men with globozoospermia compared to fertile subjects (at a ratio of ATG7/GAPDH 3.1 ± 0.94 vs. 0.71 ± 0.2; P=0.04). However, the ratio of LC3II/LC3I shows no significant difference between the two groups (0.07 ± 0.04 vs. 0.06 ± 0.03; P=0.8) (Fig .2). 

In this study, the correlations between measured parameters and markers in total population were analyzed as depicted in Table 1. The results showed significantly negative associations of sperm concentration and motility with the expression of ATG7 (P<0.05). Besides, a significantly positive correlation was found between the percentage of DNA fragmentation and expression of ATG7 (P<0.05). There were also significant negative correlations among the percentage of DNA fragmentation, sperm concentrations, and motility (P<0.05).

**Table 1:** Correlations between autophagy markers, semen parameters and DNA damage.


Parameters	ATG7	LC3II/LC3I	Concentration (10^6^/ml)	Motility (%)	Abnormal sperm morphology (%)	TUNEL (%)

**ATG7**	1	-0.223	-0.679^*^^*^(P=0.005)	-0.640^*^ (P=0.01)	0.421	0.841^*^ (P=0.018)
**LC3II/LC3I**	-0.223	1	-0.302	-0.204	0.232	-0.179
**Concentration(10^6^/ml)**	-0.679^*^^*^ (P=0.005)	-0.302	1	0.722^*^^*^ (P<0.001)	-0.207	-0.663^*^(P= 0.026)
**Motility (%)**	-0.640* (P=0.01)	-0.204	0.722^*^^*^ (P<0.001)	1	-0.316	-0.693^*^(P= 0.018)
**Abnormal sperm morphology (%)**	0.421	0.232	-0.207	-0.316	1	0.264


*; Correlation is significant at the 0.05 level (2-tailed), **; Correlation is significant at the 0.01 level (2-tailed), ATG7; Autophagy-related 7, and TUNEL; Terminal deoxynucleotidyl transferase dUTP nick end labeling.

## Discussion

Globozoospermia is a type of primary infertility in men with a prevalence of less than 0.1% among all types of male infertilities (1). Recent studies demonstrated that autophagy is involved in acrosomal biogenesis, and testis specific inactivation of Atg7 leads to deformation of the acrosome (8,9). Therefore, we aimed to analyze some markers involved in autophagy in spermatozoa of individuals with globozoospermia. The present study demonstrated that the expression of ATG7 was significantly higher in globozoospermic individuals compared to the control group. However, no difference was found in LC3II/LC3 expression between the two groups. According to a study conducted by Lee et al., to induce autophagy and achieve acrosome biogenesis, the LC3 portein has to be deacetylated by Sirt1, allowing the de-acetylated LC3 protein to be transferred from the nucleus to the cytoplasm (13). Regarding our obtained results, it is possible that due to defects in the expression of other genes involved in the acrosomal biogenesis, such as DPY19L2 (5-7), spermatozoa increase the activation of autophagy which is perceptible by the increased expression of ATG7. However, because of the deletion of DPY19L2 gene, the acrosome biogenes does not completely occur. Our findings also suggest that autophagy is initiated but not completed, as we observed no change in a ratio of LC3II/LC3 expression. The confirmation of this hypothesis requires further studies.

Considering that autophagy may be induced in the form of cell death (14), another reason for the increase in ATG7 expression in inefertile men with globozoospermia may be an increased rate of cell death. In this regard, higher sperm DNA fragmentation, as one of the causes of cell death and chromatin damage, has been reported in these individuals (2, 15, 16) even in Dpy19l2- deficient globozoospermic spermatozoa (17). On the other hand, autophagy normally helps cells survive in stressful conditions; however it can ultimately result in cell death. Also, a previous study demonstrated a low fertilization rate in men with higher 10% thresholds, as measured by the TUNEL assay (18). In this regard, we also observed that the mean sperm DNA fragmentation was higher than 10% in infertile men with globozoospermia compared to fertile men. Therefore, we suggested that one of the possible cause of failure in assisted reproduction techniques in these individuals may be owing to the high percentage of sperm DNA fragmentation in the semen samples. 

The observed significant negative correlations among ATG7 expression, sperm concentration, and motility in the present study suggest that in some sperm cells, autophagy and cell death are completed, led to the reduced sperm concentrations. Moreover, a significantly positive correlation between sperm DNA fragmentation and the expression of ATG7, as well as a significantlt positive association between abnormal sperm chromatin packaging (protamine deficiency) and the expression of ATG7 (19) may confirm the activation of the ATG7 pathway, as a promoter of cell death. Regardless of a significantly negative correlation between sperm motility and the expression of ATG7 (20) in this study, our results were consistent with findings obtained in a study carried out Zhang et al. who showed that the sperm motility of zebrafish would be improved upon the inhibition of autophagy (21). These differences between studies may be related to sample size ,species ,and type of samples.

## Conclusion

Sperm samples from globozoospermic men show high expression of ATG7 which would be expected to remove a number of abnormal spermatozoa, as defined by chromatin abnormal packaging and/or damaged DNA in this condition. A significantly negative correlation among sperm concentration, and motility, and the expression of ATG, along with a significantly positive correlation among sperm DNA fragmentation, the sperm parameters, and the expression of ATG7 might be linked with the activation of the autophagy pathway, as a compensatory mechanism for driving deficient spermatozoa to undergo cell death. Autophagy can act as a double-edged sword that, at the physiological level, is involved in acrosome biogenesis, while, at the pathological events, such as globozoospermia and varicocele it possibly acts as a compensatory mechanism for promoting deficient spermatozoa to undego cell death. One of the limitations of this study was small sample size. Therfore,further studies are needed to confirm this hypothesis in different pathologies of male infertility. 

## References

[1] Dam AH, Feenstra I, Westphal JR, Ramos L, van Golde RJ, Kremer JA (2007). Globozoospermia revisited. Hum Reprod Update.

[2] Perrin A, Coat C, Nguyen MH, Talagas M, Morel F, Amice J (2013). Molecular cytogenetic and genetic aspects of globozoospermia: a review. Andrologia.

[3] Hotaling JM, Smith JF, Rosen M, Muller CH, Walsh TJ (2011). The relationship between isolated teratozoospermia and clinical pregnancy after in vitro fertilization with or without intracytoplasmic sperm injection: a systematic review and meta-analysis. Fertil Steril.

[4] De Braekeleer M, Nguyen MH, Morel F, Perrin A (2015). Genetic aspects of monomorphic teratozoospermia: a review. J Assist Reprod Genet.

[5] Elinati E, Kuentz P, Redin C, Jaber S, Vanden Meerschaut F, Makarian J (2012). Globozoospermia is mainly due to DPY19L2 deletion via non-allelic homologous recombination involving two recombination hotspots. Hum Mol Genet.

[6] Kuentz P, vanden Meerschaut F, Elinati E, Nasr-Esfahani MH, Gurgan T, Kilani Z (2013). Assisted oocyte activation overcomes fertilization failure in globozoospermic patients regardless of the DPY19L2 status. Hum Reprod.

[7] Khawar MB, Gao H, Li W (2019). mechanism of acrosome biogenesis in mammals. Front Cell Dev Biol.

[8] Wang H, Wan H, Li X, Liu W, Chen Q, Wang Y (2014). Atg7 is required for acrosome biogenesis during spermatogenesis in mice. Cell Res.

[9] Liu C, Song Z, Wang L, Yu H, Liu W, Shang Y (2017). Sirt1 regulates acrosome biogenesis by modulating autophagic flux during spermiogenesis in mice. Development.

[10] Nakatogawa H (2013). Two ubiquitin-like conjugation systems that mediate membrane formation during autophagy. Essays Biochem.

[11] Cao XW, Lin K, Li CY, Yuan CW (2011). A review of WHO laboratory manual for the examination and processing of human semen (5th edition). Zhonghua nan ke xue=(National Journal of Andrology).

[12] Zhang M, Jiang M, Bi Y, Zhu H, Zhou Z, Sha J (2012). Autophagy and apoptosis act as partners to induce germ cell death after heat stress in mice. PLoS One.

[13] Lee IH, Cao L, Mostoslavsky R, Lombard DB, Liu J, Bruns NE (2008). A role for the NAD-dependent deacetylase Sirt1 in the regulation of autophagy. Proc Natl Acad Sci USA.

[14] Yonekawa T, Thorburn A (2013). Autophagy and cell death. Essays Biochem.

[15] Hosseinifar H, Yazdanikhah S, Modarresi T, Totonchi M, Sadighi Gilani MA, Sabbaghian M (2015). Correlation between sperm DNA fragmentation index and CMA 3 positive spermatozoa in globozoospermic patients. Andrology.

[16] Eskandari N, Tavalaee M, Zohrabi D, Nasr-Esfahani MH (2018). Association between total globozoospermia and sperm chromatin defects. Andrologia.

[17] Yassine S, Escoffier J, Martinez G, Coutton C, Karaouzene T, Zouari R (2014). Dpy19l2-deficient globozoospermic sperm display altered genome packaging and DNA damage that compromises the initiation of embryo development. Mol Hum Reprod.

[18] Simon L, Liu L, Murphy K, Ge S, Hotaling J, Aston KI (2014). Comparative analysis of three sperm DNA damage assays and sperm nuclear protein content in couples undergoing assisted reproduction treatment. Hum Reprod.

[19] Foroozan-Broojeni S, Tavalaee M, Lockshin RA, Zakeri Z, Abbasi H, Nasr-Esfahani MH (2019). Comparison of main molecular markers involved in autophagy and apoptosis pathways between spermatozoa of infertile men with varicocele and fertile individuals. Andrologia.

[20] Aparicio IM, Espino J, Bejarano I, Gallardo-Soler A, Campo ML, Salido GM (2016). Autophagy-related proteins are functionally active in human spermatozoa and may be involved in the regulation of cell survival and motility. Sci Rep.

[21] Zhang J, Zhang X, Liu Y, Su Z, Dawar FU, Dan H (2017). Leucine mediates autophagosome-lysosome fusion and improves sperm motility by activating the PI3K/Akt pathway. Oncotarget.

